# Communities of Practice in Acute and Forensic Psychiatry: Lessons Learned and Perceived Effects

**DOI:** 10.1007/s11126-021-09923-w

**Published:** 2021-06-09

**Authors:** Sylvia Gerritsen, Anne Laura Van Melle, Lieke Johanna Cornelia Zomer, Guy Antoine Marie Widdershoven, Yolande Voskes

**Affiliations:** 1 Department of Ethics, Law and Humanities, Amsterdam University Medical Centers, VU University F-Wing, De Boelelaan 1089a, 1081 HV Amsterdam, Netherlands; 2 GGZ inGeest, Amsterdam, The Netherlands; 3 GGz Breburg, Tilburg, the Netherlands; 4 Tranzo Scientific Center for Care and Wellbeing, Tilburg School of Social and Behavioral Sciences, Tilburg University, Tilburg, Netherlands

**Keywords:** Practice Development, Mental Health Care, Forensic Mental Health, Quality of Care, Interprofessional Practice, Audit

## Abstract

In the Netherlands, two new approaches have been developed for acute and forensic psychiatry, called High and Intensive Care (HIC) and Forensic High and Intensive Care (FHIC). The models provide standards for temporary high-quality clinical care for patients in crisis and combine practices to reduce seclusion. To support the implementation of these approaches, Communities of Practice (CoPs) were created, including peer providers, mental health nurses, psychiatrists and managers. CoPs are increasingly used in healthcare. However, CoPs vary greatly in form and objective, and more insight is needed in the organisation and facilitation of CoPs. Therefore, the aim of this study is to gain insight into the lessons learned and perceived effects of the CoPs. A qualitative approach was used. Data were collected through focus groups (n = 3) with participants in the CoPs, feedback meetings with teams implementing HIC (n = 78) or FHIC (n = 23), and observations by the researchers. Data were analysed thematically. Lessons learned are: 1) create an ambassador role for CoP participants, 2) organize concrete activities, 3) take care of a multidisciplinary composition, and 4) foster shared responsibility and work on sustainability. Perceived effects of the CoPs were: 1) support of HIC and FHIC implementation, 2) creation of a national movement, and 3) further development of the HIC and FHIC approaches. The audits served as an important vehicle to activate the CoPs, and stimulated the implementation of HIC and FHIC. The findings may help others in creating a CoP when it comes to the implementation of best practices and improving healthcare.

## Introduction

In recent years, two new care approaches have been developed in the Netherlands: High and Intensive Care (HIC), which focusses on acute psychiatry [1] and Forensic High and Intensive Care (FHIC), which focusses on forensic psychiatry (*Werkboek Fhic. High en Intensive Care vanuit forensisch perspectief,* 2017). Both approaches have been formulated as care models, based on a comprehensive set of best- and evidence-based practices to support care professionals and institutions to intensify care in case of a crisis, and prevent and reduce the use of coercive measures. Currently, the care models are being implemented nationwide in Dutch (forensic) mental healthcare institutions [2, 3].

To support and stimulate care organization with the implementation of HIC and FHIC, two Communities of Practice (CoPs) were created. The creation of the CoPs aimed to facilitate interaction and learning among care professionals in (forensic) mental healthcare in order to foster the implementation of HIC and FHIC. CoPs are according to Wenger [4]: “groups of people who share a concern or a passion for something they do and learn how to do it better as they interact regularly”.

The CoPs of HIC and FHIC are national groups composed of care professionals working at different wards implementing HIC or FHIC. A core activity of the CoPs was performing site visits to each other’s institution by organizing audits [2]. In addition, participants in the CoPs regularly gathered at national meetings to exchange experiences and knowledge. In this way a structured interaction among care professionals from a large number of Dutch (forensic) mental healthcare institutions was created.

Within the healthcare sector, CoPs are becoming more popular [5, 6]. However, there is great variety in the form and objective of CoPs [6]. Also, more insight is needed in how to facilitate CoPs [7]. The aim of this study is to gain insight into lessons learned from the CoPs of HIC and FHIC, and into perceived effects. This was investigated through qualitative research focusing on the perspective of the auditors, audit-receiving teams and observations made by the researchers. The findings may help others in creating a CoP when it comes to the implementation of best practices and the improvement of healthcare.

## Background

### The HIC and FHIC models

The HIC and the FHIC models provide standards for temporary high-quality clinical care for patients in crisis and combine evidence based interventions and best practices to reduce coercion. When outpatient care is not sufficient due to crisis, the patient will be temporarily admitted to a HIC or FHIC [1]. The HIC model was developed first based on former research and through meetings of professionals, peer providers and family representatives [1]. Forensic institutions were interested in a similar approach and through several expert meetings and research the FHIC model was developed, with a central focus on safety in contact and an open institutional climate (*Werkboek Fhic. High en Intensive Care vanuit forensisch perspectief,* 2017).

In both care models, the emphasis is on restoring and maintaining contact, risk assessment and crisis prevention through stepped care [1]. This stepped care is visible in the combination of the 'high care function' (HC) and the 'intensive care function' (IC). The moment the patient cannot stay on the regular closed ward (High Care) with other patients, care can be temporarily scaled up to the IC, where intensive care units (ICUs) and High Security Rooms (HSRs) are located. When a patient is transferred to the IC, a nurse will accompany the patient to provide one-to-one guidance. Key elements of the HIC and the FHIC model are hospitality, healing environment and the extensive collaboration with outpatient or other referring care services, patients and their relatives [1].

### The CoPs of HIC and FHIC

#### The formation of the CoPs

In the development of HIC and FHIC, many mental healthcare institutions and professionals from acute and forensic psychiatry were involved. Based on former experiences on reduction of coercion, we decided to bring (mental) healthcare professionals together and involve them to jointly learn and reflect [8]. For this reason, we created CoPs of professionals working on the implementation of HIC and FHIC. This resulted in two national groups composed of multidisciplinary care professionals from 26 mental healthcare institutions for HIC, and 16 forensic mental healthcare institutions for FHIC.

To further stimulate exchange and cooperation, a fidelity scale (the HIC monitor) was developed to assess compliance to the model [2]. For FHIC, a comparable process took place which resulted in the FHIC monitor. To foster the implementation of HIC and FHIC audits were organized, in which the degree of adherence to the model was assessed by scoring the monitor. Therefore, a group of representative care professionals from each institution was formed. Participating institutions selected a number of care professionals from different disciplines, including nurses, psychiatrists, social workers and managers. In the CoP of FHIC also peer providers participated, as they are part of the FHIC team and were expected to bring a valuable perspective from their own experience as patient. All care professionals received a 1-day training to be able to perform audits. For HIC a total of 50 care professionals participated within the period of 2014 and 2018. For FHIC a total of 37 care professionals participated in the period of 2017 and 2019. Together these care professionals had a central position with the CoPs of HIC and FHIC.

#### Activities of the CoP

The core activity of the care professionals within the CoPs of HIC and FHIC was the performance of audits. During an audit, two or three trained auditors from different institutions visited a ward from another institution. In this way, professionals from different institutions were brought together, which facilitated the sharing of knowledge and experiences. During an audit, auditors received a tour through the ward, held interviews with team members and patients, observed a team meeting and performed a file check. Based on this information, auditors scored the model fidelity scale for either HIC or FHIC. Three times a year, meetings took place with the auditors to update their knowledge, learn from each other and discuss experiences obtained during audits.

Various additional activities were organized besides the audits. Meetings were organized that brought the participants of the CoPs together, including other professionals as policy makers and researchers. Starting from 2013, yearly HIC conferences were organized, in which research, newly developed interventions, innovations, treatment procedures and local projects in line with HIC and FHIC were presented. In addition, informal platform meetings for HIC and FHIC were organized on a yearly basis. In this way, a platform for care workers was provided to discuss challenges and opportunities to work with the HIC and FHIC approaches the care models in daily practice. During the conferences and platform meetings, the participants of the CoPs played an active role in presentations and workshops.

#### The Role of The Researchers as Facilitators

For both CoPs, the researchers had a facilitating role. They supported exchange of knowledge and experiences by planning and organizing trainings, follow up meetings with the CoP, audits and focus groups with HIC and FHIC teams. In addition, they were involved in the development of the program and organization of the yearly conferences and informal platform meetings.

## Methods

### Aim

The aim of this study was to gain insight into the process of creating the CoPs of HIC and FHIC and its specific lessons learned, and in the perceived effect of the CoPs from the perspective of auditors and teams.

### Design

To gain insight in the CoPs that were created to support the implementation process of HIC and FHIC into practice, a qualitative approach was used. By using focus groups and feedback meetings, viewpoints, perspectives and experiences were exchanged in a dynamic and interactive way [9]. The focus groups and feedback meetings followed a semi-structured design by using a supportive guide, and addressed lessons learned and perceived effects of the CoPs. The focus groups and feedback meetings were facilitated by four researchers. In addition, observations were made by the researchers. Findings are reported in line with the Consolidated criteria for Reporting Qualitative research (COREQ) checklist [10].

### Participants

Participants in this study were care professionals of the mental health and forensic institutions. Institutions were approached for participation if they were in the process of implementing HIC or FHIC. In this article we will refer to auditors, by which we mean the trained care professionals who performed HIC or FHIC audits at other care institutions. In addition, we will refer to teams, by which we mean the teams working on the implementation of HIC and FHIC and received an audit in this context. Both the group of auditors as teams were diverse in disciplines, work experience, gender and age. Many participants were mental health nurses.

### Data Collection

During three follow up meetings with auditors, data were collected through focus groups to create insight in their views and experiences of performing audits. Additionally, data was extracted from feedback meetings with teams after each audit. Also, observations by the researchers were used as data.

1. *Focus groups with auditors*
  A total of three focus groups were organized at a central location in the Netherlands; two focus groups with HIC auditors and one focus group with FHIC auditors. The goal of these focus groups was to evaluate the process of performing audits and reflect upon the experiences of the auditors. The focus groups lasted approximately 90 min.
  During the first focus group, organized in April 2015, with 20 HIC auditors, the auditors were asked to describe 1) three positive experiences with regard to the audit process and 2) aspects they have learned from the process of performing audits and what was of added value for their own institutions. The answers on these questions were written down on post-its and gathered on large flip-overs, as input for the plenary discussion on these topics.
  The second focus group was organized in October 2017 with a group of 13 FHIC auditors. Auditors were asked to describe 1) positive experiences with regard to the audit process, 2) challenges with regard to the audit process and 3) the aspects they have learned from the process and brought to their own institution. Answers on these questions were gathered on flip-overs as input for a plenary discussion.
  During the third focus group with 16 HIC auditors in March 2018, participants were asked to reflect upon their experiences of performing HIC audits. The group of HIC auditors was divided into small groups of four to six people. They were asked to develop a vacancy advertisement to recruit new auditors, using their own experiences for the text. The vacancy had to include 1) the work field of auditors, 2) the profile of an ideal auditor 3) the benefits of being an auditor and 4) the downsides of being an auditor.
2. *Feedback meetings with teams*
  After each audit, a feedback meeting was organized led by the researchers, with the team which was audited to reflect on the audit scores. Next to this, the aim of the meeting was to evaluate the process and effects of the audits. In total, 78 feedback meetings were conducted with HIC teams and for FHIC 23 feedback meetings took place. Over the years, half of the teams received two audits and, as a result, participated in the feedback meetings twice. The researchers visited the teams at their institution. In order to include multiple perspectives, in each participating team, members from at least three different disciplines (e.g. nurses, social workers, peer providers, psychiatrist, psychologist or managers) were included. On average, five team members attended the feedback meetings. The feedback meetings lasted approximately 120 min.
3. *Observations from the researchers*
  Data was also derived from observations made by the researchers. Insights could therefore be included about the way care professionals in the CoP interact and profiled themselves, and how they were perceived by others. The researchers discussed the observations and used this in comparison with the data derived from the focus groups and feedback meetings.
  The focus group discussions, feedback meetings and observations were audio-recorded and transcribed verbatim, or field notes were made by the researchers. Furthermore, the notes were supported by documentary evidence (flip-overs of the focus groups with auditors).

### Ethical Considerations

Participating institutions received an information letter about the study, and prior to each focus group and feedback meeting the researchers explained the study to its participants and asked them to give verbal consent. On site, a number of participants were unable to participate due to practical reasons for example because of situations at the ward in which they were needed to help. To prevent data from being traceable to persons or institutions, identifiable data have been coded. The Medical Ethical Committee of the researchers institution approved the study.

### Data Analysis and Rigour

The data retrieved from the focus groups and feedback meetings were analysed in MAXQDA version 2018, using a thematic analysis and a coding tree [11]. In this process, the researchers also assessed the saturation on data. First, the data was labelled with codes, as part of an open coding approach, performed by three researchers. When doubt existed, codes were discussed. Second, for each research question, codes were clustered into themes that matched the content of the codes. These themes were discussed among four researchers until consensus was reached (investigator triangulation; [12]). By using this predefined categories but also allowing themes to emerge from the data, the thematic coding combined a deductive and inductive approach [13]. To check the researchers interpretations, a member check was performed for each focus group and feedback meeting [14]. A small remark was sent by a number of participants, though this did not result in adjustments in the analysis. The final themes were discussed with two researchers from the research group who were not involved in the data collection or the formation of the CoPs to ensure objectivity.

## Results

This section presents the findings of the study. Based on the analysis of the qualitative data, themes were identified regarding (A) *lessons learned* in the CoPs; and (B) *perceived effects* of the CoPs. The study participants worked in acute or forensic psychiatry at wards located throughout the country, and varied in gender, work experience, age, and discipline.

### A. Lessons Learned

From the data the following lessons learned were identified**:** 1) create an ambassador role for CoP participants, 2) organize concrete activities, 3) take care of a multidisciplinary composition, and 4) foster shared responsibility and sustainability.

#### Create an Ambassador Role for CoP Participants

Within their own institutions, auditors were seen and approached as substantive experts on the (F)HIC model. A nurse of an audited team indicated:

*“One of the coordinating nurses focuses on the HIC model and, in his role as HIC auditor, he offers an additional source of knowledge regarding the model.”*

Colleagues expect from auditors to take the lead in developments regarding HIC or FHIC. Because of the contact and exchange between auditors and care professionals from other institutions, auditors were familiar with national developments. They acquired an ambassador role within their own institution with regard to the implementation of HIC and FHIC. This ambassador role was for many auditors something to be proud of. Auditors indicated that they also positioned themselves as an expert and took an exemplary role for colleagues. As a HIC auditor mentioned:

*“A positive experience is that my role as auditor gives me an expert position within my institution.”*

Professionals were proud to be an auditor and this was visible on social media and in their email signatures, where they specifically indicated to be a (F)HIC auditor.

From this, the following lesson can be derived: participants in CoPs can find inspiration and acknowledgement in having an ambassador role for the new care approaches.

### Organize Concrete Activities

Participants considered the audits as an important vehicle to foster active exchange among care professionals in acute and forensic psychiatry. As mentioned by one of the HIC auditors:

*“The audits offer an occasion for exchange of knowledge between institutions, in which ideas can also be gained for your own institution. It is better to take over something good then to invent something bad.”*

Next to the audits, national meetings provided an opportunity for learning from others. A HIC team expressed that they would like to gather experiences about a best practice during a national meeting:

*“We envision the feasibility of one of the best practices as a major challenge. Therefore, we would like to obtain experiences from other HIC wards where this is already well organized. Possibly this would be an interesting theme for the coming HIC platform meeting.”*

This results in the following lesson: the organization of concrete activities can foster energy and active exchange among CoP participants.

### Take Care of a Multidisciplinary Composition

The CoPs of HIC and FHIC consisted of people with a variety of disciplines, such as nurses, social workers, psychologists, psychiatrists and managers. Therefore, the CoPs did not include a one-sided perspective and teams receiving audits indicated to feel understood and represented. As indicated by a FHIC team:

*“All three auditors had different backgrounds which made the conversation interesting. During the day many aspects were recognized and at the same time the auditors were pleasantly surprised about what they heard.”*

Care professionals were able to ask questions on the basis of their own expertise. Combining and sharing ideas from different disciplines, each with their own views, experiences and perspectives, enriched the CoPs. Additionally, it was considered crucial that auditors worked at a HIC or FHIC ward themselves. When providing feedback during an audit, auditors were able to relate to their own working environment.

In the FHIC CoP, the perspective of peer providers was highly valued during audits, as they were able to ask critical questions from their own experience as being patients. An audit-receiving team mentioned:

*“The auditors were passionate and we were particularly impressed by the peer provider. This auditor highlighted the person behind the patient, something that we experienced as confronting but very valuable. We want to take his advice and feedback along, for example by just looking at the naming of something: are you talking about a cell or a bedroom?”*

This leads to the following lesson: taking care of a multidisciplinary composition can strengthen the exchange of knowledge and experience among participants.

### Foster Shared Responsibility and Work on Sustainability

As participants in the CoPs, auditors felt responsible for the continuation of the implementation of HIC and FHIC, both on a national level and in their own institution. In the assignment during a focus group to write a text for a vacancy for a position in the audit team, HIC auditors noted:

*“As auditor you have the responsibility to take on a pioneering role with regard to HIC in your own institution; you set an example to colleagues and take them along in the process.”*

Auditors constantly engaged colleagues of their own institution in the developments, by sharing insights and inviting them to national meetings. In this way, the gap in knowledge, enthusiasm and responsibility between care professionals was diminished. If this does not happen right from the start, there is a risk that colleagues of auditors will feel insufficiently included in the ongoing developments, as indicated by a FHIC team:

*“At the moment it feels like the train has already started to run and the team is now being thrown on it instead of starting to run with the team in it.”*

Care professionals also took care to actively involve their care institution in the CoPs, by approaching management and asking for support. A participant in the FHIC CoP said:

*“We hope to get a reasonable amount of time [from the institution] to motivate and guide the entire team. This is a fundamental aspect to be able to implement FHIC”.*

Participants in the CoPs also mentioned the risk of frequent staff changes at clinical wards. A member of a FHIC team said:

*“There has been and will be many changes in our team so we have to make sure that the FHIC approach does not disappear from sight.”*

This requires a shared responsibility and careful transfer of knowledge and roles within the CoP. To foster sustainable CoPs, the initial facilitators and all care professionals should together take responsibility and initiative.

From this, the following lesson can be learned: the continuity of implementation requires sharing the responsibility and work on sustainability.

### B. Perceived Effects

Based on the analysis, three perceived effects of the CoPs came to the fore: 1) support of HIC and FHIC implementation, 2) creation of a national movement and 3) further development of the HIC and FHIC approaches. These effects are further explained in the sections below.

#### Support of HIC and FHIC Implementation

All care professionals perceived that the CoPs had an effect on the implementation of HIC and FHIC. For instance by reflecting on work routines and the exchange of experiences, as a HIC auditor said:

*"It is helpful to put one's own working routines under a magnifying glass and at the same time exchange experiences with regard to these routines."*

In this process, it was experienced as helpful to compare ways of working on similar wards in other parts of the country. This could also result in the awareness of being distinctive or good at something, and provide an example for other institutions. Because of this, auditors and teams felt proud. Sometimes, the CoP even resulted in a competitive feeling, as a FHIC auditor mentioned in the focus group:

*“It makes you competitive, and you are more aware of pitfalls that you see at other institutions.”*

Furthermore, auditors and teams mentioned that as a result of the exchange within the CoP barriers could also be discussed and overcome. To hear or see that other professionals were able to implement the new approach was perceived as helpful. An example is a forensic care institution which envisioned FHIC as difficult to implement due to their strong focus on security. After seeing and hearing about a best practice during a self-organized site visit, they were surprised about the feasibility of this intervention. A nurse explains:

*“During a site visit, situations were sketched out that we had not thought to be possible to do at our own institution. It became clear that we could implement this intervention as well.”*

So hearing and seeing how other care professionals at similar wards work creates confidence among care professionals regarding the possibility to overcome difficulties of implementing HIC or FHIC.

From this, the following perceived effect can be derived: the CoPs were experienced as a means to support implementation of HIC and FHIC.

### Creation of a National Movement

The CoPs participants often mentioned a feeling of togetherness and ownership of HIC or FHIC. Auditors felt part of a large national movement regarding HIC or FHIC. This was confirmed by audit-receiving teams, as they appreciated the visit and input of the auditors as signs of being part of a larger movement. A FHIC team member says:

“*The audit and the auditors' visit gave energy because we realized that FHIC lives nationwide and not only within our institution.*”

Care professionals working with HIC or FHIC throughout the country were able to find each other easily, because of the contact that was established in the CoP. Next to the formal activities that were organized by the researchers, participants were able to find each other, as one of the nurses said:

*“I try to make contacts and find the right people, because I know that there are institutions that work according to this [best-practice]”.*

Because of the contact between professionals of different institutions, more collaboration was facilitated, as illustrated by a quote of one of the HIC auditors:

*“HIC and the audits created short lines between mental healthcare institutions”*

From this, the following perceived effect can be derived: the CoPs helped creating a national movement.

### Further Development of the HIC and FHIC Approaches

In the focus groups, auditors indicated that they felt responsible to contribute to the development of HIC or FHIC, and were willing to find out more about a particular subject in order to share this within the CoP. As a result of the audits, auditors were aware of new developments and proposed additions to the HIC or FHIC monitor. Working with model fidelity scales intensively during the audits allowed care professionals to give feedback on the content of the scales. Possibilities for improvements of the scales were discussed during the regular meetings with auditors. Both auditors and audited teams valued the experience that their ideas and feedback on the model were included in the development of HIC and FHIC. As one of the members of a HIC team said:

*“It is a positive experience to be heard when providing critical comments on criteria in the HIC monitor.”*

From this, the following perceived effect can be derived: the CoPs contributed to the further development of the HIC and FHIC approaches.

## Discussion

The aim of this study was to derive lessons learned from the creation of the HIC and FHIC CoPs, and gain insight into their perceived effects.

The topics mentioned in the lessons learned show similarities with the four key characteristics of CoPs mentioned in a review by Li et al. [5]: 1) social interaction, 2) knowledge-sharing, 3) knowledge-creation and 4) identity-building. National audits and training and refection days for auditors fostered interaction between care professionals, and sharing and creating of knowledge. This also spread to teams in mental healthcare institutions implementing HIC or FHIC through the audits and national meetings. The auditors had a central position in the CoPs, gaining an identity as ambassador for HIC or FHIC within their own institutions and on a national level. This can be related to the fourth characteristic of a CoP: identity-building [5].

Our results with regards to the lessons learned also show a difference with the existing literature on CoPs. We found that the multidisciplinary composition of the CoPs increased mutual understanding between disciplines and strengthened the exchange of knowledge during for instance the audits. In contrast, former research indicated that a mix of disciplines in a CoP can be a challenge because members envisioned the CoP differently or experienced a barrier to participate in conversations about care [15, 16]. When members of a CoP do not feel included or heard this can result in an unsafe and non-learning environment [17]. An explanation for our different findings might be the presence of a shared vision, in this case the HIC or FHIC approach. Another explanation might be that the training of the auditors suppressed possible hierarchy between disciplines and made care professionals more equal.

The CoPs fostered implementation of HIC and FHIC, and created a national movement. This also means that the CoPs stimulated quality improvement and reduction of coercive measures. This is relevant given that the reduction of seclusion in psychiatry is challenging and requires ongoing practice development [18]. This finding confirms the shifting aim of CoPs; from learning and exchange towards changing or improving practice [7, 19, 20]. This reality change was closely related to the use of audits in both CoPs. The audits were experienced as a means to inspire and support each other in the implementation of HIC and FHIC. Auditors structured the site visits by using the model fidelity scale. These findings are consistent with research from Bindels et al. [15], indicating: “the importance of co creating rules of interaction with CoP members and a structured method appreciated by all to foster each other’s input”. Combing and structuring various elements within a CoP, for instance by organizing audits, might foster the creation of a CoP and a reality change.

### Strengths and Limitations

This study shows a number of strengths and limitations. The strengths include the variety in participants, setting and data collection. Two comparable CoPs from different settings were included, as well as a large number of care institutions and multidisciplinary care professionals. Although the study was performed in both acute and forensic psychiatry, it is unknown whether the findings are generalizable for other (healthcare) sectors and countries. The role of the researchers as CoP facilitators can be considered as both a strength and limitation. On the one hand, the close involvement may have helped to deepen the analysis, while on the other hand it may have hindered a more distanced analysis. To limit this, two researchers with a less active role in the CoPs facilitation were involved.

## Conclusion

To conclude, this paper presented lessons learned and perceived effects of two CoPs within acute and forensic mental health care. Lessons learned regarded the importance of an ambassador role for CoP participants, of organizing concrete activities, of assembling expertise of professionals with various backgrounds, and of fostering shared responsibility and work on sustainability. The perceived effects of the CoPs included fostering implementation of HIC and FHIC, creating a national movement, and contributing to the further development of the HIC and FHIC approach. Specifically, the audits served as an important vehicle to activate the CoPs, and stimulated the implementation of HIC and FHIC.

## Data Availability

The datasets used and analysed during the current study are available from the corresponding author on reasonable request.
